# Correlation of Standardized Uptake Value and Apparent Diffusion Coefficient in Integrated Whole-Body PET/MRI of Primary and Recurrent Cervical Cancer

**DOI:** 10.1371/journal.pone.0096751

**Published:** 2014-05-07

**Authors:** Johannes Grueneisen, Karsten Beiderwellen, Philipp Heusch, Paul Buderath, Bahriye Aktas, Marcel Gratz, Michael Forsting, Thomas Lauenstein, Verena Ruhlmann, Lale Umutlu

**Affiliations:** 1 Department of Diagnostic and Interventional Radiology and Neuroradiology, University Hospital Essen, Essen, Germany; 2 Department of Diagnostic and Interventional Radiology and Neuroradiology, University Hospital Duesseldorf, Duesseldorf, Germany; 3 Department of Obstetrics and Gynecology, University Hospital Essen, Essen, Germany; 4 Erwin L. Hahn Institute for Magnetic Resonance Imaging, University Duisburg-Essen, Essen, Germany; 5 Department of Nuclear Medicine, University Hospital Essen, Essen, Germany; West German Cancer Center, Germany

## Abstract

**Background:**

To evaluate a potential correlation of the maximum standard uptake value (SUV_max_) and the minimum apparent diffusion coefficient (ADC_min_) in primary and recurrent cervical cancer based on integrated PET/MRI examinations.

**Methods:**

19 consecutive patients (mean age 51.6 years; range 30–72 years) with histopathologically confirmed primary cervical cancer (n = 9) or suspected tumor recurrence (n = 10) were prospectively enrolled for an integrated PET/MRI examination. Two radiologists performed a consensus reading in random order, using a dedicated post-processing software. Polygonal regions of interest (ROI) covering the entire tumor lesions were drawn into PET/MR images to assess SUV_max_ and into ADC parameter maps to determine ADC_min_ values. Pearson’s correlation coefficients were calculated to assess a potential correlation between the mean values of ADC_min_ and SUV_max_.

**Results:**

In 15 out of 19 patients cervical cancer lesions (n = 12) or lymph node metastases (n = 42) were detected. Mean SUV_max_ (12.5±6.5) and ADC_min_ (644.5±179.7×10^−5^ mm^2^/s) values for all assessed tumor lesions showed a significant but weak inverse correlation (R = −0.342, p<0.05). When subdivided in primary and recurrent tumors, primary tumors and associated primary lymph node metastases revealed a significant and strong inverse correlation between SUV_max_ and ADC_min_ (R = −0.692, p<0.001), whereas recurrent cancer lesions did not show a significant correlation.

**Conclusions:**

These initial results of this emerging hybrid imaging technique demonstrate the high diagnostic potential of simultaneous PET/MR imaging for the assessment of functional biomarkers, revealing a significant and strong correlation of tumor metabolism and higher cellularity in cervical cancer lesions.

## Introduction

Cervical cancer is the third most commonly diagnosed cancer and the fourth leading cause of cancer death in females worldwide [1]. An overall recurrence rate of up to 30% and an overall 5-year survival rate of 73% have been reported, substantially depending on the initial local tumor extent and potential metastatic tumor spread [2]. Hence, accurate initial staging and high-quality patient follow-up are mandatory to provide best possible patient- and therapy management.

Magnetic resonance imaging (MRI) has been established as a valuable imaging modality for the assessment of cervical tumor extent and potential metastatic lesions [3]. Aside from morphologic imaging, diffusion weighted imaging (DWI) has been widely established in oncological imaging. DWI has been shown to be a highly sensitive imaging method for lesion detection (primary tumors, nodal and distant metastases) as well as for lesion characterization, based on the quantification of the apparent diffusion coefficient (ADC) [4], [5], [6]. The ADC allows for quantification of diffusivity, which is shown to be restricted in the majority of malignancies due to higher cellularity compared to surrounding tissue. Multiple studies have already demonstrated a high correlation among reduced ADC values of different tumor entities as well as an association to tumor aggressiveness, risk of metastases and/or tumor recurrence [7], [8], [9].

Within the last decade, 18F-fluoro-deoyglucose positron emission tomography (^18^F-FDG-PET) has been predominantly applied as a part of hybrid imaging (PET/CT), combining morphological and functional data, thus achieving an increase in sensitivity for tumor staging and the determination of recurrent malignancies [10], [11], [12], [13]. In clinical practice, PET/CT has been established as a valuable imaging technique for detection and treatment monitoring of tumor lesions, providing additional data about the metabolic activity of tumor lesions. The strongest metabolic uptake is represented by the maximum standard uptake value (SUV_max_). SUV_max_ is considered the most commonly applied parameter to quantify the tracer uptake, yielding strong association to tumor aggressiveness and to patient prognosis [14], [15], [16].

Previous trials revealed an inverse correlation among metabolic uptake and restricted diffusivity in diverse cancer entities, based on SUVs derived from PET/CT data sets and ADC values derived from subsequent MR data sets [17], [18], [19]. The recent introduction of integrated PET/MRI systems has provided the basis for correlation of simultaneously derived SUV and ADC measurements, potentially yielding a reduction of co-registration and motion artifacts as well as the omission of ionizing CT radiation.

Hence, the aim of this study was to evaluate a potential correlation of SUV_max_, reflecting the highest metabolic activity, and ADC_min_, representing the highest tumor cellularity, in primary and recurrent cervical cancer based on integrated PET/MRI examinations.

## Materials and Methods

### Patients

The University Hospital Essen ethics committee authorized the examinations as part of fundamental single center research on integrated PET/MRI. 19 consecutive patients (mean age 51.6 years; range 30–72 years) with histopathologically confirmed primary cervical cancer (n = 9) or suspected tumor recurrence (n = 10) were prospectively enrolled in our study. Tumor recurrences were determined based on physical examination (n = 3) during clinical follow-up and abnormal findings in CT or MR imaging (n = 7). A whole-body integrated PET/MRI examination was performed after written informed consent was obtained from all patients.

### PET/MRI

Whole-body PET/MRI examinations were performed on a Magnetom Biograph mMR (Siemens Healthcare Sector, Erlangen, Germany), using lutetium oxyorthosilicate-based avalanche photodiodes (APD) for PET-acquisition. PET/MRI scans were obtained with an average delay of 102±39 min after ^18^F-FDG injection and a mean activity of 201±69 MBq. Whole body PET scans, covering the body from skull-base to mid-thighs, were performed with 5 to 6 bed positions and an acquisition time of 8 minutes each. PET images were reconstructed using the iterative algorithm OSEM, 3 iterations and 21 subsets, Gaussian filter with 4 mm full width at half maximum (FWHM) and a 344×344 image matrix. PET data were automatically attenuation corrected using a four-compartment-model attenuation map (µ-map) calculated from fat-only and water-only as obtained from Dixon-based sequences. Simultaneous whole-body MR imaging was performed with a dedicated mMR head and neck coil and phased-array body surface coils. The overall examination time of the PET/MR protocol for whole-body staging amounted to 35±5 min., encompassing the following sequences:

- A coronal 3-dimensional volume interpolated breath-hold examination (VIBE) sequence with a slice thickness of 3.12 mm (repetition time [TR], 3.6 milliseconds; echo time [TE], 1.23 and 2.46 milliseconds; flip angle, 10.0 degrees; FOV, 500 mm; phase FOV, 65.6% for Dixon-based attenuation correction),
- A transverse diffusion-weighted echo-planar imaging (EPI) sequence in free breathing with a slice thickness of 5.0 mm (TR, 9900 milliseconds; TE, 82 milliseconds; b-values: 0, 500 and 1000 s/mm^2^, matrix size 160×90; FOV, 420 mm, phase FOV, 75%; GRAPPA, acceleration factor 2),
- A coronal 2-dimensional turbo inversion recovery sequence magnitude (TIRM) sequence with a slice thickness of 5.0 mm (TR, 3190 milliseconds; TE, 55 milliseconds; matrix size 384×216; FOV, 450 mm; phase FOV 75% GRAPPA, acceleration factor 2),
- A transversal 2-dimensional fat-saturated half Fourier acquisition single-shot turbo spin echo (HASTE) sequence with a slice thickness of 5 mm (TR, 1500 milliseconds; TE, 117 milliseconds; matrix size 320×211; FOV, 450 mm; phase FOV, 81.3%; GRAPPA, acceleration factor 2),
- Pelvis only: A transversal 3-dimensional VIBE sequence with a slice thickness of 2.5 mm (TR, 4.46 milliseconds; TE, 1.71 milliseconds; matrix size 512×230; FOV, 300 mm, phase FOV 68.8%, GRAPPA, acceleration factor 2),
- Pelvis only: A sagittal 3-dimensional VIBE sequence with a slice thickness of 2.5 mm (TR, 4.46 milliseconds; TE, 1.71 milliseconds; flip angle, 9.0 degrees; matrix size 512×246; FOV, 300 mm; phase FOV 68.8% GRAPPA, acceleration factor 2). For dynamic imaging three repetitive scans were acquired at a delay of 20, 60 and 90 seconds after the application of i.v. contrast agent (0.1 mmol/kg bodyweight of 1 M-Gadobutrol, Bayer Healthcare, Germany),
- Pelvis only: A sagittal turbo-spin echo (TSE) sequence with a slice thickness of 4.0 mm (TR, 4440 milliseconds; TE, 101 milliseconds; matrix size 512×221; FOV, 280 mm; phase FOV, 71.9%; GRAPPA, acceleration factor 2),
- A transversal whole-body 3-dimensional VIBE sequence with a slice thickness of 3.5 mm (TR, 4.08 milliseconds; TE, 1.51 milliseconds; matrix size 512×230; FOV, 400 mm; phase FOV, 75%; GRAPPA, acceleration factor 2).

For contrast-enhanced imaging, 0.1 mmol/kg Gadobutrol (Gadovist, Bayer HealthCare, Germany), followed by a saline flush of 20 ml, was injected intravenously at 2 ml/s using an automated injector (Spectris solaris EP MR Injection System, Medrad, Germany).

### Image Analysis

Two radiologists with 11 and 7 years of experience in reading MRI and hybrid imaging, respectively, performed a consensus reading in random order, using a post-processing software (OsiriX 5.0.2). For image analysis only cervical cancer lesions and lymph node metastases were included, whereas metastatic or recurrent lesions of other localizations where excluded from analysis. To determine the SUV_max_, tumor margins of primary and recurrent cervical cancer lesions and lymph node metastases were identified on T1w MR imaging and a polygonal region of interest (ROI) was manually placed on every slice within the tumor on fused PET/MRI images covering the entire target lesion.

To investigate a potential correlation between the SUV and ADC values, an ADC map was generated by the scanner software (syngo MR B18P, Siemens, Erlangen, Germany) using three b-values (b = 0 s/mm2, b = 500 s/mm^2^, b = 1000 s/mm^2^). All tumor lesions were identified on diffusion-weighted sequences (b = 0) and a ROI was manually drawn encompassing the entire target lesion. After automatic transfer and visual confirmation of correct placement onto the corresponding parameter map the ADC_min_ was determined.

### Reference Standard

Histopathological confirmation of primary cervical cancer lesions was available in all 9 cases. Histopathological confirmation of each lymph node metastasis and recurrent tumor lesion was not reasonable, particularly in patients where surgical procedures were not indicated according to patient management guidelines. Therefore, in accordance with prior publications, [20], [21] a consensus characterization for each depicted lesion was performed, taking into account all available histopathological samples, prior examinations, PET/MRI examinations and clinical follow-up imaging.

### Statistical Analysis

Statistical analysis was performed using the IBM SPSS version 21 (SPSS Inc, Armonk, NY, USA). Data are expressed as mean values ± standard deviation (SD). Descriptive analysis was used to assess SUV and ADC values. To indicate potential significant differences between the SUV_max_ and ADC_min_ for primary and recurrent tumor lesions, Wilcoxon’s signed-rank was utilized. Pearson’s correlation coefficient were calculated to assess a correlation between the SUV_max_ and ADC_min_ values. According to the classification system provided by Salkin, r values between 0.8 and 1.0 represent a very strong correlation, between 0.6 and 0.8 a strong correlation, between 0.4 and 0.6 a moderate correlation and between 0.2 and 0.4 a weak correlation. Values between 0.0 and 0.2 are classified as showing a weak or no relationship [22]. A p-value <0.05 was considered to indicate statistical significance.

## Results

All 19 patients with primary (n = 9) and recurrent (n = 10) cervical cancer successfully completed the whole-body PET/MRI examinations without any relevant side effects. PET/MRI enabled correct identification of a total of 60 cancerous lesions, comprising 12 cervical cancer lesions (9 primary tumors and 3 local recurrences) and a total of 42 lymph nodes metastases in 15 patients. Four out of the enrolled 19 patients showed tumor recurrences in other locations than the cervix or lymph nodes [peritoneal carcinosis (n = 4), liver metastasis (n = 1) and bone metastasis (n = 1)], and thus were excluded from data analysis.

Mean values for SUV_max_ (12.5±6.5) for all assessed tumor lesions exhibited a weak but significant inverse correlation (R = −0.342, p<0.05) with the corresponding ADC_min_ values (644.5±179.7×10^−5^ mm^2^/s, Table 1).

**Table 1:** Correlations of the maximum Standardized Uptake Value (SUV_max_) and the minimum Apparent Diffusion Coefficient (ADC_min_).

	(a) All lesions	(b) Primary Recurrent		(c) Cervical cancer Lymph nodes	
	SUV_max_	SUV_max_	SUV_max_	SUV_max_	SUV_max_
**ADC_min_**	−0.342	−0.692	−0.136	−0.612	−0.246
**p-value**	0.011	0.001	0.451	0.035	0.116

Column a shows correlations for all tumor lesions. Column b shows correlations after subdivision into primary versus recurrent tumor lesions and column c yields correlations after subdivision into cervical cancer lesions and lymph node metastases.

**Figure 1:**
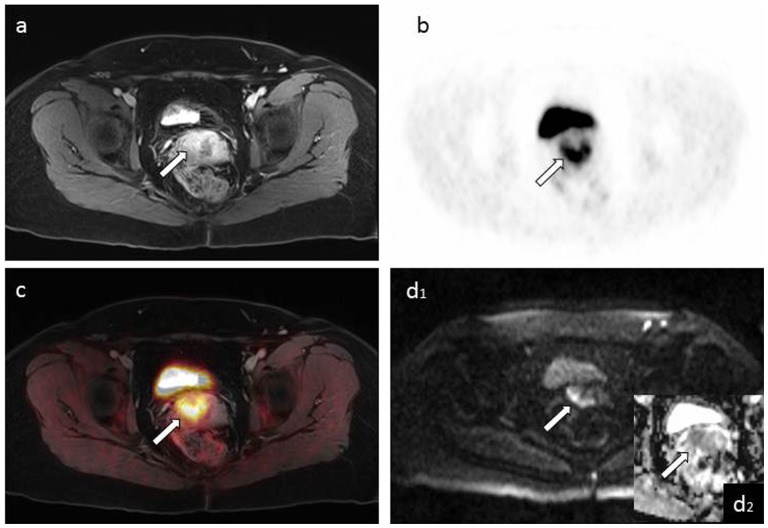
Figure a shows a T1w 3D VIBE image of an inhomogeneously contrast-enhancing mass lesion of the cervix of a 49-year-old patient. Corresponding PET (b) and fused PET/MRI images (c) demonstrate elevated FDG-uptake. The lesion shows corresponding diffusion impairment in DWI (d_1,_ b = 1000) and low signal intensity in the ADC map (zoom image d_2_).

**Figure 2:**
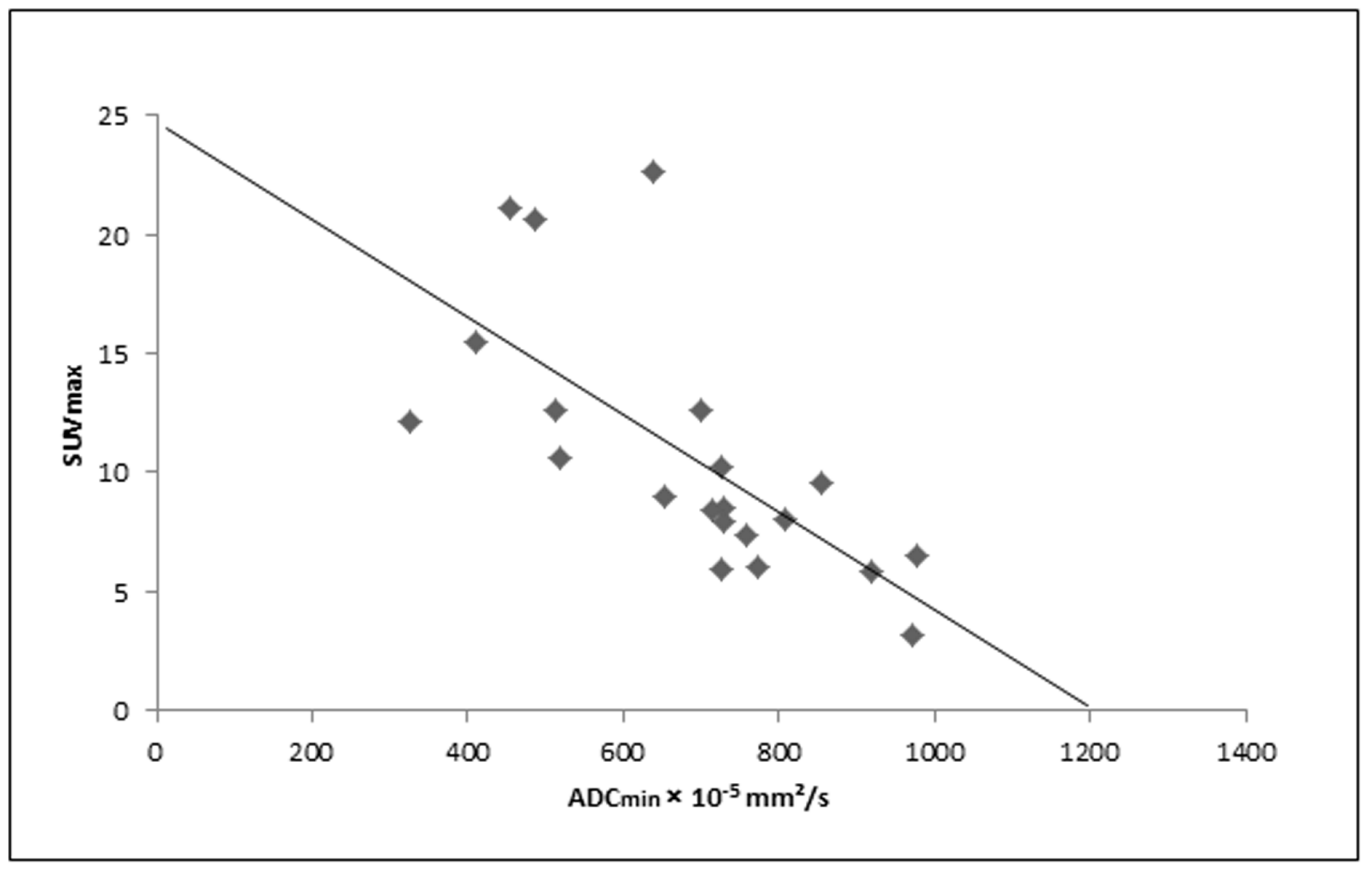
Scatter plot demonstrating a significant inverse correlation between the ADC_min_ and the SUV_max_ for primary cervical cancer lesions and associated lymph node metastases. (n = 21; R = −0.692; p<0.05).

When subdivided into primary and recurrent cancer lesions (including cervical lesions and lymph nodes), primary tumors (Figure 1) and associated primary lymph node metastases revealed a strong inverse correlation between SUV_max_ and ADC_min_ (R = −0.692, p<0.001; Figure 2), while recurrent cancer lesions (recurrent cervical lesions and lymph node metastases) did not show a significant correlation. Furthermore, the results revealed a non-significant tendency of lower SUV_max_ and higher ADC_min_ values for tumor recurrences in comparison to primary tumor lesions (Table 2).

**Table 2:** Correlations of ADC_min_ and SUV_max_ of primary and recurrent cancer lesions, demonstrating a tendency of higher ADC_min_ and lower SUV_max_ values for recurrent cancers.

	ADC_min_ (×10^−5^ mm^2^/s)	SUV_max_
**Primary**	618.9±177.8	13.7±6,9
**Recurrent**	684.6±160.6	10.7±5.3
**p-value**	0.181	0.520

**Figure 3:**
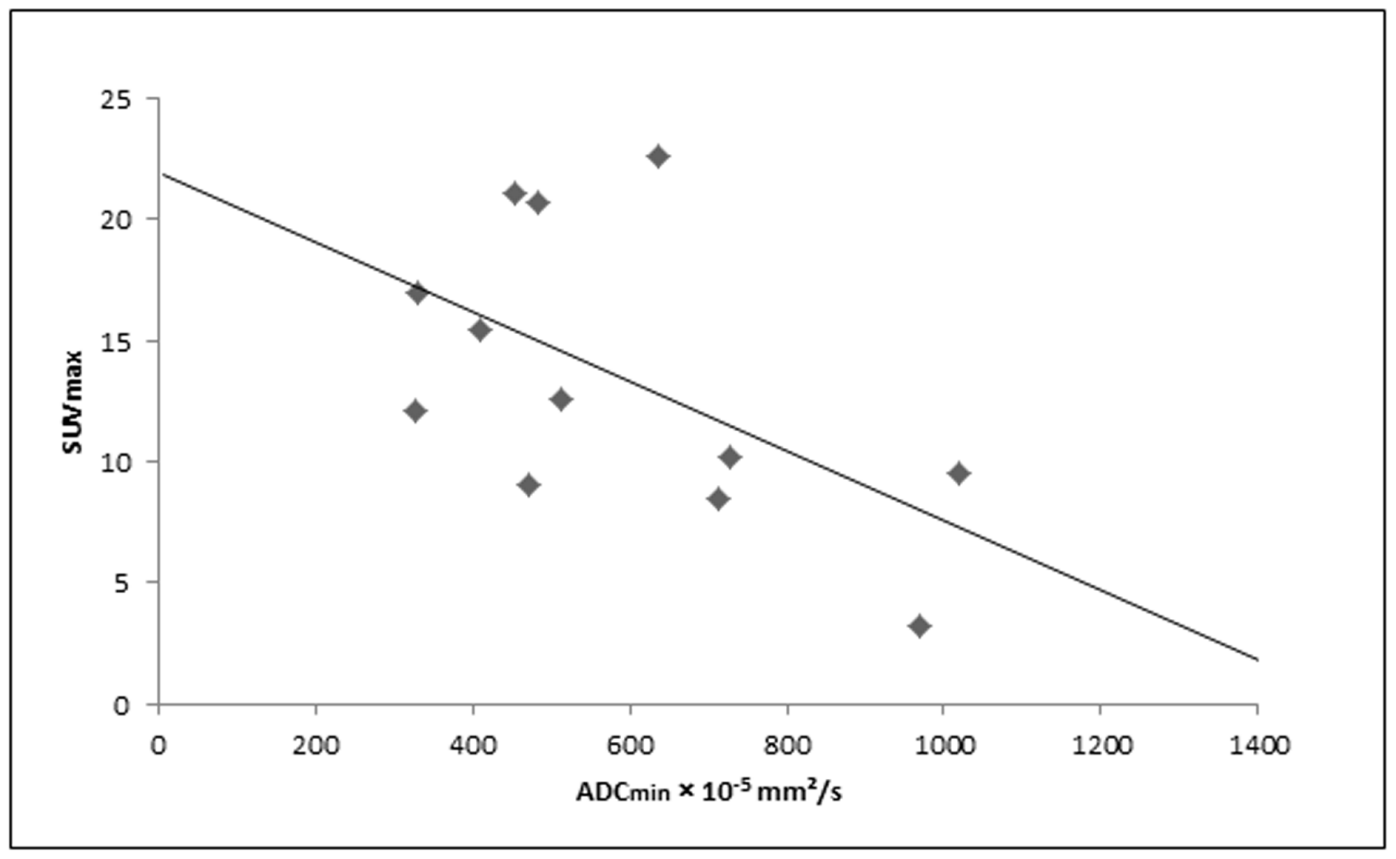
Scatter plot showing a significant inverse correlation between the ADC_min_ and the SUV_max_ for primary and recurrent cervical cancer lesions. (n = 12; R = −0.628; p<0.05).

When subdivided into cervical lesions and lymph node metastases, cervical lesions exhibited a strong inverse correlation (n = 12; R = −0.612, p<0.05; Figure 3), while SUV_max_ and ACD_min_ values for lymph node metastases (n = 42) did not reveal a significant correlation.

## Discussion

The successful implementation of integrated PET/MRI systems has enabled a new platform for simultaneous assessment of MRI and PET based functional parameters. Simultaneous acquisition of morphologic and functional data, in terms of PET and DWI, bears the potential to offer highly-accurate co-registration and reduction of motion and misregistration artifacts. This may be particularly valuable for investigations of the female pelvis, as different patient positioning and varying filling of the bladder (and respectively varying positioning of the uterus) as well as movement of surrounding intestinal tissue may lead to difficulties in registration of the corresponding functional and morphologic datasets [23]. Furthermore, fast repetitive dynamic imaging is an established part of the imaging protocol for cervical cancer, also demanding simultaneous hybrid imaging for clean co-registration. Another beneficial matter of simultaneous PETMRI lies in the significant reduction of time in comparison to subsequent PET and MRI data acquisition. Initial studies on integrated hybrid imaging have demonstrated the general feasibility of this new imaging technique as well as the stable reproducibility and comparability of PET/MRI derived SUVs to SUVs derived from PET/CT datasets [21], [24], [25], [26].

Within the last decade molecular imaging, by means of DWI and PET/CT, has gained an important status in the diagnostic workup of tumor disease and the evaluation of potential metastatic tumor spread. The implementation of DWI in oncologic imaging has been proven to be useful for an improvement in tumor characterization, cancer detection, outcome prediction and treatment monitoring [27], [28]. Based on restricted motion of water molecules within cancer lesions caused by higher cell densities, ADC values derived from DWI have been demonstrated to be inversely correlated to cellularity, yielding lower ADC values for various malignancies compared to surrounding healthy, inflammatory or scar tissue [29], . Hence, DWI and quantification via ADC have been recognized as a useful diagnostic adjunct in oncologic imaging.

Although the basic principle of tumor detection in DWI and PET is based on different aspects of tumor biology, both modalities overlap in their clinical applications in terms of tumor detection and characterization. While DWI is based on assessment of potential diffusion restriction, PET enables an evaluation of tumor metabolism. Rapid proliferation of tumor cells leads to changes in cell metabolism to guarantee sufficient nutrition supply. Previous studies could show an association between increased expression levels of proteins of the glycolysis pathway (e.g. GLUT1) and an elevated uptake of the glucose in tumor cells [31], [32]. Therefore, an accumulation of the radiotracer-marked glucose, in terms of 18F-fluoro-deoyglucose, indicates an increased metabolic activity of viable tumor cells which can be quantified by SUV measurements. Since SUV_mean_ measurements are affected by the threshold of tumor boundaries, thus leading to a variability of the values, the more robust maximum standardized uptake values (SUV_max_) are commonly used in the majority of studies [33]. Comparable to restricted diffusion, elevated maximum SUVs are associated to malignancy and can be utilized for assessment of therapy response under chemotherapy [34], [35].

Numerous publications have revealed a correlation between tissue metabolism, quantified in SUVs, and tissue cellularity, quantified in ADC values, derived from PET/CT and subsequent MRI datasets for diverse tumor entities, including cervical cancer [17], [19], [36], [37]. Both functional parameters have been shown useful predictors for treatment response and survival [14], [34], [37], [38]. While many studies could reveal an inverse correlation among SUV_max_ and ADC_min_, a number of studies revealed varying correlations among SUV_max_ and absolute/relative ADC values [24]. Gu et al examined patients with rectal cancer and showed a significant negative correlation between SUV_max_ and ADC_min_ as well as SUV_mean_ and ADC_mean_ [36]_._ Investigating a potential correlation in cervical cancer, Ho et al enrolled a total of 33 patients with primary cervical cancer prior to radiation or chemotherapy [33]. Their results revealed an inverse correlation between SUV_max_ and the ratio of ADC_min_ and ADC_mean_, yet not between the absolute values of SUV_max_ and ADC_min_. Nakamura et al assessed the correlation of both biomedical markers to cervical cancer-related features [37]. The authors predicted a shorter overall and disease-free survival in patients with cervical cancer exhibiting higher SUV_max_ in combination with lower ADC_min_ values.

While in the majority of previous studies, ADC and SUV measurements were derived from subsequent PET/CT and MR examinations, the introduction of integrated PET/MR scanners has enabled a new platform for simultaneous DWI and PET data acquisition. Due to the very recent implementation of integrated hybrid scanner systems, the amount of published data investigating a potential correlation between simultaneously obtained SUV and ADC values is restricted to a few tumor entities, excluding cervical cancer up to current status. Nevertheless, initial studies revealed a correlation among standardized uptake values derived from PET/CT and PET/MRI examinations, underlining the diagnostic competence of SUV derived from PET/MRI, despite the different principles of attenuation correction performed for both hybrid imaging techniques [24], [25], [39]. As MRI is not associated with the radiodensity of the examined tissue, attenuation correction (AC) is performed utilizing a well-recognized MR-suitable method based on the segmentation of attenuation map into 4 classes, first introduced by Martinez-Möller [40].

**Figure 4:**
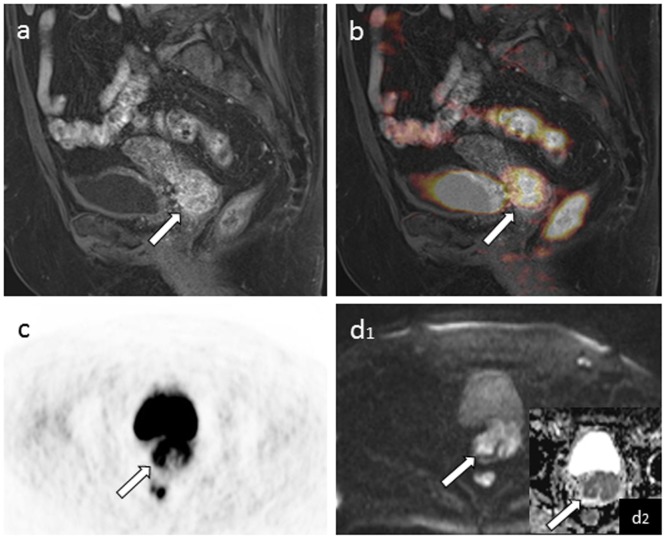
T1 weighted post-contrast sagittal 3D VIBE (a) and fused PET/MRI image (b) of an inhomogeneously contrast-enhancing and FDG avid cervical cancer lesion. Image c and d show corresponding tracer uptake in PET (c) and restricted diffusion in DWI (d_1_) with low signal intensity on the corresponding ADC map (d_2_).

To the best of our knowledge, this study is the first publication to investigate a potential correlation of SUV and ADC values in patients with primary and recurrent cervical cancer derived from integrated PET/MRI examinations. As SUV_max_ and ADC_min_ are considered to represent the most proliferative part or the highest cellularity within a tumor lesion, we chose to investigate a potential correlation. Our study results reveal a significant inverse correlation between the SUV_max_ and the ADC_min_ in patients with primary cervical cancer lesions and corresponding lymph nodes (Figure 4) and a weak, yet non-significant correlation for recurrent tumor lesions (including cervical cancer lesions and lymph nodes). This disparity among primary and recurrent cancers may be due to therapy-induced microstructural changes in cellularity and metabolism of recurrent cancer, leading to a decrease of SUV and increase of ADC values.

Our results are in line with previous publications on primary cervical cancers, in terms of revealing a general correlation of SUV and ADC values. Yet, in contrary to the publication of Ho et al, only revealing a significant correlation of the ratio of SUV_max_ and ADC_min_, our data analysis showed a direct significant correlation of the two parameters [33]. This controversial correlation between the two biomarkers has been well-recognized in numerous trials, investigating diverse tumor entities and may be caused by a number of altering conditions [41], [42]. One reason for differing correlations may lie in the timing of the performed PET and MRI examinations. Retrospectively evaluated, some study protocols exhibited varying time intervals between the two examinations, ranging from immediately subsequent PET/CT and MRI to a 2-week interval [43], [44]. In contrary to the subsequent data acquisition presented by Ho et al, in our study both datasets were acquired simultaneously on an integrated PET/MRI system, thus minimizing potential misregistration artifacts and potential biological changes. Furthermore, ADC quantifications are known to be highly susceptible to general physical alterations based on the choice of b-values, tissue perfusion, scanner geometry and field strength [45],[46].

Despite displaying the great diagnostic potential of this new emerging imaging technique, the present study is not without limitations. Based on the limited number of patients, our results should be considered preliminary and further studies with larger patient cohorts are required to strengthen these initial findings. Secondly, histopathological confirmation was available for all primary cervical cancer lesions, however, guideline-based patient management did not comprise surgical procedures or histopathological confirmation of all recurrent cancers. Hence, in accordance with previous publications [20], [21] a modified reference standard was utilized, taking into account all available histopathological specimens, prior examinations, clinical follow-up and all cross-sectional follow up imaging. Furthermore, a direct comparison of the SUVs derived from PET/MRI to SUV derived from PET/CT would have been desirable. Yet, our study refers to previous publications, demonstrating the high diagnostic potential of SUV derived from integrated PET/MRI exclusively, as well as to publications, revealing the high correlation among SUV derived from PET/CT in direct comparison to SUV derived from subsequent integrated PET/MRI [24], [25].

In conclusion, presenting the first data on simultaneous PET/MRI imaging of patients with cervical cancer, our study underlines the strong diagnostic potential of this emerging hybrid imaging technique for simultaneous assessment of functional biomarkers, revealing a significant correlation of tumor metabolism and a higher cellularity in cervical cancer lesions.
